# A systematic review and meta-analysis comparing the safety and efficacy of open oesophagectomy and hybrid minimally invasive oesophagectomy

**DOI:** 10.1308/rcsann.2025.0066

**Published:** 2025-09-04

**Authors:** N Naim, MDS Reza

**Affiliations:** James Paget University Hospitals NHS Foundation Trust, UK

**Keywords:** Open oesophagectomy, Hybrid minimally invasive oesophagectomy, Oesophageal cancer, Conduit necrosis, Anastomotic leak, Chyle leak

## Abstract

**Introduction:**

Surgical resection, with or without neoadjuvant therapy, remains the primary treatment for oesophageal cancer. The two main surgical approaches are open oesophagectomy (OE) and hybrid minimally invasive oesophagectomy (HMIE). However, their relative safety and efficacy remain controversial. This review aims to compare perioperative and postoperative complications between OE and HMIE in the management of oesophageal cancer.

**Methods:**

Web of Science, EMBASE, PubMed, Scopus and the Cochrane Library were searched for relevant studies. Odds ratios (OR), standard mean differences (SMD) and 95% confidence intervals (CI) were used for statistical analysis.

**Results:**

Eight studies involving 6,053 patients were included. HMIE was associated significantly with lower rates of conduit necrosis (risk ratio (RR)=3.54, 95% CI [1.07, 11.73]; *p*=0.04), postoperative pneumonia (RR=1.29, 95% CI [1.05, 1.57]; *p*=0.01) and recurrent laryngeal nerve paralysis (RR=2.51, 95% CI [1.13, 5.55]; *p*=0.02). No significant differences were observed in Clavien–Dindo complication grades IIIa–IVb (RR=1.13, 95% CI [0.92, 1.38]; *p*=0.24), grade V complications (RR=1.03, 95% CI [0.30, 3.51]; *p*=0.96), bleeding, inhospital mortality, 90-day mortality, duration of surgery, hospital stay or intensive care unit stay. Although not statistically significant, OE was associated with fewer cases of anastomotic and chyle leaks.

**Conclusions:**

Both OE and HMIE have distinct advantages and drawbacks. HMIE appears superior in reducing conduit necrosis, postoperative pneumonia and nerve paralysis, whereas OE has slightly lower rates of anastomotic and chyle leaks. Surgical approach should be tailored to individual patient profiles. Further studies are needed to assess long-term oncologic outcomes.

## Introduction

Oesophageal cancer is the tenth most common cancer globally, accounting for approximately 1 in 18 cancer-related deaths.^1^ For patients with tumors localised within the oesophagus or lymph nodes, oesophagectomy – either with or without neoadjuvant therapy – remains the standard treatment option.^2^ However, oesophagectomy is associated frequently with significant morbidity and mortality due to postoperative complications.^3–5^

The two-phase Ivor Lewis procedure is the approach used most commonly in the United Kingdom.^2^ To reduce complication rates, minimally invasive techniques have increasingly been adopted.^6^ Compared with open oesophagectomy (OE), minimally invasive oesophagectomy (MIE) aims to minimise tissue trauma and promote faster recovery. MIE techniques include:

• Hybrid minimally invasive oesophagectomy (HMIE): a laparoscopic approach is used for either the abdominal or thoracic phase.
• Totally minimally invasive oesophagectomy (TMIE): both abdominal and thoracic phases are performed laparoscopically.

Recent studies suggest MIE provides superior perioperative outcomes and comparable survival benefits relative to OE.^7,8^ This systematic review compares the safety and efficacy of OE and HMIE.

## Methods

This systematic review followed the Cochrane Handbook for Systematic Reviews of Interventions and adhered to PRISMA guidelines.^9,10^ The study was registered in PROSPERO (CRD420251032279) and structured using the PICO (Population, Intervention, Comparison, Outcome) framework.

### Study selection criteria

Eligible studies included randomised controlled trials (RCTs) and prospective or retrospective cohort studies. Excluded were:

• Non-English publications
• Letters to the Editor
• Animal studies
• Studies with unreliable data

Two independent reviewers screened the studies using predefined inclusion and exclusion criteria.

### Risk of bias assessment

The Cochrane Risk of Bias 2 (ROB-2) tool was used to assess methodological quality in RCTs.^11^ Studies were categorised as having ‘low’, ‘some concerns’, or ‘high’ risk of bias.

### Data extraction

Data were extracted into Excel spreadsheets and included the following parameters:

• Study ID
• Country and design
• Intervention type
• Patient demographics
• Outcomes categorised as:
  - o Postoperative complications
  - o Perioperative metrics
  - o Mortality rates

Postoperative outcomes assessed included Clavien–Dindo complications (grades IIIa–Vb), anastomotic and chyle leaks, conduit necrosis, pneumonia, recurrent laryngeal nerve paralysis and bleeding. Perioperative metrics included duration of surgery, hospital stay and intensive care unit (ICU) stay. Mortality outcomes included inhospital and 90-day mortality.

### Statistical analysis

Data analysis was performed using RevMan version 5.4. Risk ratios (RR) were used for dichotomous variables and mean differences (MD) for continuous outcomes. Heterogeneity was assessed using the *I*² statistic and chi-square test. A fixed-effects model was applied unless heterogeneity necessitated a random-effects model.

## Result

### Selection of studies

An initial search of five databases yielded 2,555 studies. After removing duplicates, 1,323 articles were screened. Based on title and abstract review, 1,246 studies were excluded. Of the remaining 77, we excluded 3 retracted articles, 15 involving unrelated procedures, 14 with different study designs, 16 editorials and 21 review articles. Finally, eight studies met the inclusion criteria for this systematic review.^2,12–18^

**Figure 1:**
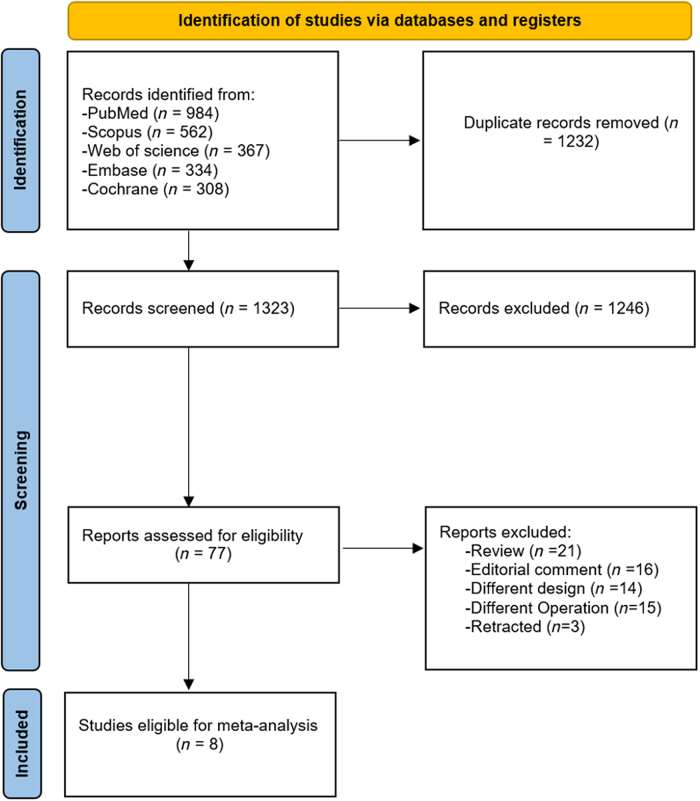
PRISMA flow diagram PRISMA = preferred reporting items for systematic reviews and meta-analyses

A PRISMA flow diagram illustrating the selection process is shown in Figure 1.

### Characteristics of included studies

The eight studies included involved a total of 6,053 patients and were conducted in Sweden, Austria, the UK, Germany, China and South Korea. The majority of participants were male, and the mean age ranged from 56 to 66 years. These studies were a mix of prospective, retrospective and RCTs. Table 1 summarises the characteristics of the included studies.

**Table 1:** Summary of included studies

Study ID	Design	Area	Time	Population	Intervention	Population by intervention	Male	Female	Age
Klevebro *et al*^12^ 2020	Prospective	Sweden	2020	246	OE	93	74	19	65.8 (29.9–82.0)
					HMIE	75	68	7	67.1 (46.3–83.2)
Schoppmann *et al*^13^ 2010	Retrospective	Austria	2010	62	OE	31	21	10	58.6 (33.7–76.8)
					MIE	31	25	6	61.5 (35.7–74.8)
Wilk *et al*^14^ 2022	Retrospective	IESG database	2022	4,733	OE	1,897	1,565	332 (18)	65 (58–71)
					HMIE	1,364	1,123	241 (18)	64 (57–71)
Metcalfe *et al*^2^ 2024	RCT	UK	2024	533	OE	266	227	39	66 (9)
					HMIE	267	225	42	67 (9)
Reichert *et al*^15^ 2020	Retrospective	Germany	2020	143	OE	105	86	19	64 (40–86)
					HMIE	38	33	5	62.5 (42–77)
Yun *et al*^16^ 2017	Retrospective	South Korea	2017	115	OE	62	61 (98.4)	1 (1.6)	68 [45–79]
					HMIE	53	51 (96.2)	2 (3.8)	66 [48–83]
Chen *et al*^17^ 2021	Retrospective	China	2021	195	OE	75	65 (86.7)	10 (13.3)	58.19 ± 7.82
					HMIE	120	94 (78.3)	26 (21.7)	56.76 ± 6.48
Paireder *et al*^18^ 2018	RCT	Austria	2018	26	OE	12	10 (83.3)	2 (16.7)	62.5 (49–77)
					HMIE	14	10 (71.4)	4 (28.6)	64.5 (40–75)

HMIE = hybrid minimally invasive oesophagectomy; IESG = International Esodata Study Group; OE = open oesophagectomy; RCT = randomised controlled trial; TMIE = totally minimally invasive oesophagectomy

### Study quality

**Figure 2:**
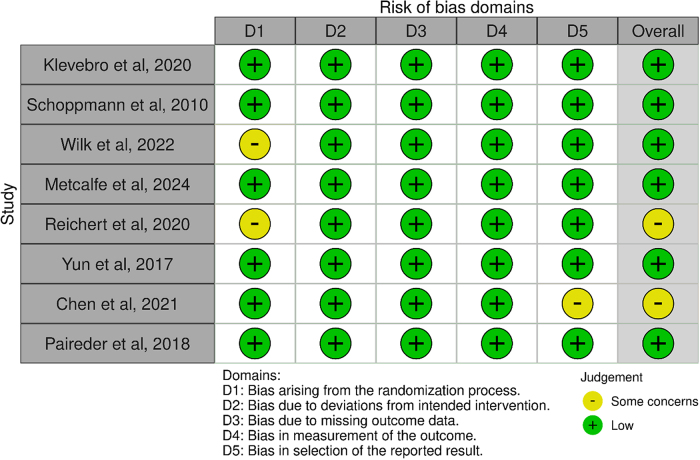
ROB-2 bias assessment tool indicating study quality ROB-2 = Cochrane Risk of Bias 2

Risk of bias was assessed using the ROB-2 tool. Figure 2 presents the traffic-light diagram summarising bias assessments. Most studies were of moderate-to-high quality, with no studies assessed as high risk overall.

### Postoperative outcomes

#### Clavien–Dindo complications (grade IIIa–IVb)

**Figure 3:**
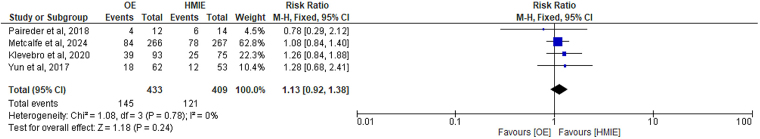
Clavien–Dindo classification of complications grade IIIa–IVb OE = open oesophagectomy; HMIE = hybrid minimally invasive oesophagectomy

Four studies (*n*=842) evaluated these complications. No statistically significant difference was found between OE and HMIE (RR=1.13, 95% CI [0.92, 1.38]; *p*=0.24), although slightly lower incidence was observed with HMIE (Figure 3).

### Clavien–Dindo complications (Grade V)

**Figure 4:**
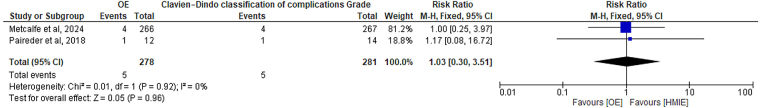
Clavien–Dindo classification of complications grade V OE = open oesophagectomy; HMIE = hybrid minimally invasive oesophagectomy

Two studies (*n*=559) assessed this outcome. The difference was not statistically significant (RR=1.03, 95% CI [0.30, 3.51]; *p*=0.96) (Figure 4).

### Anastomotic leak

**Figure 5:**
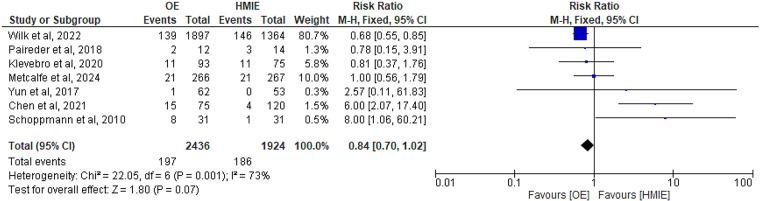
Anastomosis leak OE = open oesophagectomy; HMIE = hybrid minimally invasive oesophagectomy

Seven studies (*n*=4,360) reported this outcome. OE showed a nonsignificant trend toward fewer leaks (RR=0.84, 95% CI [0.70, 1.02]; *p*=0.07) (Figure 5).

### Chyle leak

**Figure 6:**
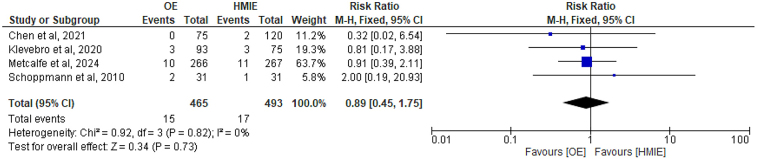
Chyle leak OE = open oesophagectomy; HMIE = hybrid minimally invasive oesophagectomy

Four studies (*n*=958) found no significant difference between OE and HMIE (RR=0.89, 95% CI [0.45, 1.75]; *p*=0.73), although slightly fewer leaks occurred in the OE group (Figure 6).

### Conduit necrosis

**Figure 7:**
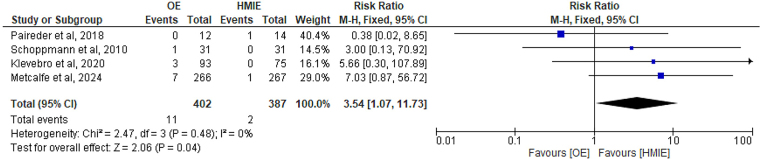
Conduit necrosis OE = open oesophagectomy; HMIE = hybrid minimally invasive oesophagectomy

Four studies (*n*=789) demonstrated a significantly higher incidence in the OE group (RR=3.54, 95% CI [1.07, 11.73]; *p*=0.04), favouring HMIE (Figure 7).

### Pneumonia

**Figure 8:**
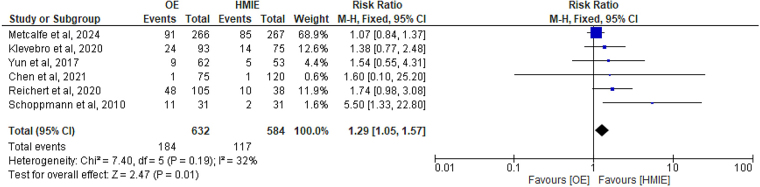
Pneumonia OE = open oesophagectomy; HMIE = hybrid minimally invasive oesophagectomy

Six studies (*n*=1,216) showed a statistically significant reduction in postoperative pneumonia with HMIE (RR=1.29, 95% CI [1.05, 1.57]; *p*=0.01) (Figure 8).

### Recurrent laryngeal nerve paralysis

**Figure 9:**
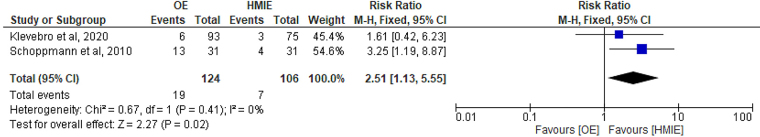
Recurrent laryngeal nerve paralysis OE = open oesophagectomy; HMIE = hybrid minimally invasive oesophagectomy

Two studies (*n*=230) reported significantly higher rates in OE compared with HMIE (RR=2.51, 95% CI [1.13, 5.55]; *p*=0.02) (Figure 9).

### Bleeding

**Figure 10:**
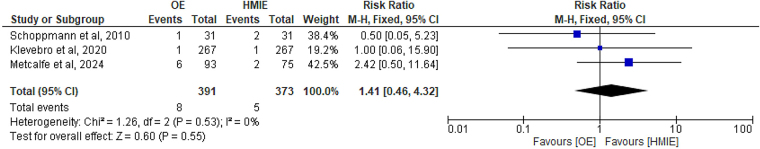
Bleeding OE = open oesophagectomy; HMIE = hybrid minimally invasive oesophagectomy

Three studies (*n*=764) showed no significant difference (RR=1.41, 95% CI [0.46, 4.32]; *p*=0.55), although HMIE showed a slightly lower rate (Figure 10).

### Perioperative outcomes

#### Duration of surgery

**Figure 11:**
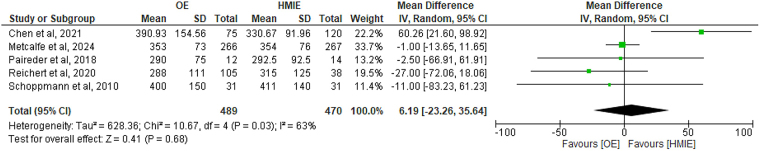
Duration of surgery in minutes OE = open oesophagectomy; HMIE = hybrid minimally invasive oesophagectomy

Five studies (*n*=959) reported slightly shorter operative times in the HMIE group (MD=6.19min, 95% CI [–23.26, 35.64]; *p*=0.68), although not statistically significant (Figure 11).

### Hospital stay

**Figure 12:**
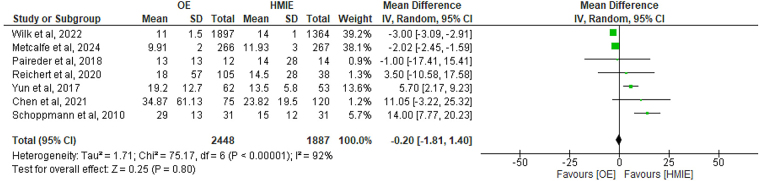
Hospital stays in days OE = open oesophagectomy; HMIE = hybrid minimally invasive oesophagectomy

Five studies (*n*=4,335) showed no significant difference (MD=–0.20 days, 95% CI [–1.81, 1.40]; *p*=0.80). The OE group had marginally shorter hospital stays (Figure 12).

### ICU stay

**Figure 13:**
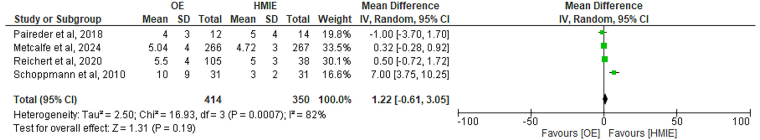
ICU stays in days OE = open oesophagectomy; HMIE = hybrid minimally invasive oesophagectomy

Four studies (*n*=764) found no significant difference (MD=1.22 days, 95% CI [–0.61, 3.05]; *p*=0.19), with HMIE slightly favoured (Figure 13).

### Mortality outcome

#### In-hospital mortality

**Figure 14:**
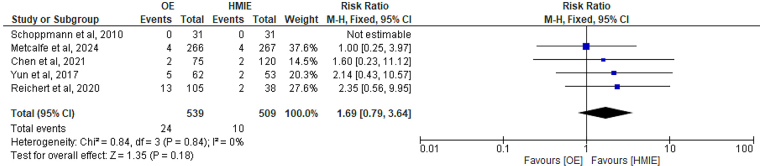
In hospital mortality OE = open oesophagectomy; HMIE = hybrid minimally invasive oesophagectomy

Five studies (*n*=1,048) showed no significant difference between OE and HMIE (RR=1.69, 95% CI [0.79, 3.64]; *p*=0.18) (Figure 14).

### 90-day mortality

**Figure 15:**
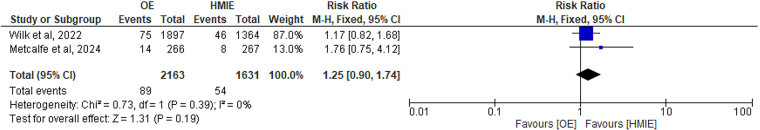
Ninety day mortality OE = open oesophagectomy; HMIE = hybrid minimally invasive oesophagectomy

Two studies (n=3,794) reported no significant difference (RR=1.25, 95% CI [0.90, 1.74]; *p*=0.19), although HMIE showed a slightly lower incidence (Figure 15).

## Discussion

This systematic review compared the safety and efficacy of OE and HMIE based on perioperative and postoperative outcomes.

Regarding postoperative complications, significant differences were observed in conduit necrosis, postoperative pneumonia and recurrent laryngeal nerve paralysis, all of which occurred less frequently in the HMIE group. These findings suggest that HMIE may offer superior postoperative safety in specific domains compared with OE. Conversely, OE was associated with a slightly lower incidence of anastomotic and chyle leaks, although these differences were not statistically significant. This suggests that, although HMIE may reduce trauma and respiratory complications, OE might offer better control over anastomotic integrity.

In terms of perioperative outcomes, HMIE was associated with marginally shorter operation time and ICU stay, which may reflect less surgical trauma and quicker immediate recovery. However, the OE group showed a slightly shorter hospital stay, indicating potential differences in postdischarge planning or institutional practices. None of these differences reached statistical significance, and therefore should be interpreted cautiously.

Mortality outcomes, both in-hospital and at 90 days postoperatively, showed no statistically significant differences between the two approaches. Although HMIE demonstrated numerically lower mortality rates, the CIs included the null value, indicating insufficient evidence to declare superiority of one technique over the other in terms of short-term mortality.

Despite the increasing adoption of minimally invasive techniques, open surgery continues to be used widely. For example, a recent study by Henckens *et al* reported that 55% of patients underwent OE, 17% HMIE and 29% TMIE.^19^ This ongoing use of OE may reflect surgeon experience, institutional preference or patient factors that limit the applicability of minimally invasive techniques.

A key strength of this review is the inclusion of multicentre, high-quality studies with largely homogeneous results. The analytical methods applied addressed heterogeneity where present. However, some outcomes lacked sufficient data for subgroup analysis, which is a notable limitation. Furthermore, potential bias in nonrandomised studies and variable definitions of outcomes across studies may have influenced the findings.

Overall, although both techniques are viable, the current evidence suggests that HMIE may offer advantages in reducing specific complications without compromising overall safety or mortality. These findings highlight the importance of individualised surgical planning, considering patient-specific factors, institutional resources and surgeon expertise.

## Conclusions

Both OE and HMIE present distinct advantages and limitations in the surgical management of oesophageal cancer. Whereas HMIE appears to offer benefits in terms of reduced postoperative complications—such as conduit necrosis, pneumonia and recurrent laryngeal nerve paralysis—OE demonstrates a slight advantage in lowering the incidence of anastomotic and chyle leaks.

In terms of perioperative factors, HMIE is associated with marginally shorter operation time and ICU stay, whereas OE is linked to a slightly shorter overall hospital stay. However, none of these differences were statistically significant. Similarly, mortality rates at discharge and within 90 days postoperatively did not differ significantly between the two techniques.

Given the narrow margins in safety and efficacy between OE and HMIE, surgical approach decisions should be individualised, taking into account patient characteristics, surgical expertise, and institutional capabilities. Further large-scale, high-quality studies are necessary to assess long-term oncologic outcomes and quality of life associated with each approach.

Ultimately, the choice between OE and HMIE should be guided by a balanced evaluation of clinical goals, patient preferences and the specific context of care.
